# Adsorption of Phenanthrene on Multi-Walled Carbon Nanotubes in the Presence of Nonionic Surfactants

**DOI:** 10.3390/ijerph20043648

**Published:** 2023-02-18

**Authors:** Huimin Cao, Zhenyang Zhou, Cuiping Wang, Hongwen Sun

**Affiliations:** Key Laboratory of Pollution Processes and Environmental Criteria, Ministry of Education, Tianjin Key Laboratory of Environmental Remediation and Pollution Control, College of Environmental Science and Engineering, Nankai University, Tianjin 300071, China

**Keywords:** phenanthrene, multi-walled carbon nanotubes, surfactants, adsorption

## Abstract

The bioavailability and mobility of phenanthrene (Phe) adsorbed by multi-walled carbon nanotubes (MWCNTs) may be substantially influenced by nonionic surfactants used both in the synthesis and dispersion of MWCNTs. The adsorption mechanisms of Phe adsorbed onto MWCNTs under the different nonionic surfactants Tween 80 (TW-80) and Triton X-100 (TX-100) in the aqueous phase were investigated in terms of changes in the MWCNTs’ compositions and structures. The results showed that TW-80 and TX-100 were easily adsorbed onto MWCNTs. Phe adsorption data onto MWCNTs were better suited to the Langmuir equation than the Freundlich equation. Both TW-80 and TX-100 reduced the adsorption capacity of Phe onto MWCNTs. When TW-80 and TX-100 were added in the adsorption system, the saturated adsorption mass of Phe decreased from 35.97 mg/g to 27.10 and 29.79 mg/g, respectively, which can be attributed to the following three reasons. Firstly, the hydrophobic interactions between MWCNTs and Phe became weakened in the presence of nonionic surfactants. Secondly, the nonionic surfactants covered the adsorption sites of MWCNTs, which caused Phe adsorption to be reduced. Finally, nonionic surfactants can also promote the desorption of Phe from MWCNTs.

## 1. Introduction

Carbon nanotubes (CNTs) have strong adsorption ability for heavy metals and persistent organic pollutants due to their high hydrophobicity and large surface area (SA); therefore, CNTs are increasingly used as adsorbents in the pollutant removal process [1,2,3,4,5]. Multi-walled carbon nanotubes (MWCNTs), a type of CNT composed of two or more sheets of carbon with a diameter range of 1–20 nm [6], have been widely applied to adsorb heavy metals [7], organic chemicals [8,9,10,11] and surfactants [12,13] from aqueous solutions. Polycyclic aromatic hydrocarbons (PAHs), such as naphthalene, phenanthrene (Phe) and pyrene, etc., were supposed to be dangerous environmental pollutants because of their severe toxicity to human health [14,15,16,17,18]. Studies have shown that Phe has carcinogenic and potential endocrine interference characteristics, and it is also a photosensitizer and mild allergen in human skin [19]. Because of their nano size, MWCNTs can enter into cells, causing damage to animals and humans. Previous studies have suggested that the toxicity of MWCNTs is not only due to their own harmful nature but also from the toxic substances sorbed by them [20]. Thus, the toxicity of MWCNTs could be further increased when adsorbing PAHs in the environment. Therefore, studying the adsorption of Phe by MWCNTs is important for controlling the release, migration and transportation of carbon nanomaterials adsorbing Phe in the environment. 

Surfactants, which are widely used as penetrants, detergents, adhesives and dispersants, as well as in flocculation, are amphiphilic compounds composed of a hydrophilic portion and a hydrophobic group [21,22]. There are a number of studies that use surfactants to disperse and stabilize MWCNTs in the aqueous phase [23,24,25]. The electrostatic/steric repulsion of surfactants could change the dispersion and aggregation of CNTs by counteracting the van der Waals attraction between MWCNTs [26]. Moreover, PAHs and nonionic surfactants are common components of sewage treatment plants; in the field of water treatment, emerging pollutant Phe and nonionic surfactants could be adsorbed by MWCNTs at the same time from aqueous media [12,13,27]. Additionally, PAHs could enter the hydrophobic core of the surfactant aggregates (micelles) formed by surfactant monomers, thus greatly improving the water solubility [28,29]. Surfactants can not only change the dispersion of MWCNTs and the solubility of organic pollutants in solution, but also change the adsorption capacity of MWCNTs to organic pollutants, which will increase the environmental risk of MWCNTs and organic pollutants [30]. However, currently, in the literature, there is no information on how different nonionic surfactants influence the adsorption of Phe on MWCNTs. Additionally, there are no data concerning the influence of nonionic surfactant concentrations on the adsorption of Phe by MWCNTs and to what extent nonionic surfactants influence the desorption of Phe from MWCNTs. Therefore, we quantitatively examined the contributions or influences of different types of nonionic surfactants on Phe-adsorbed MWCNTs, which is conducive to the use of MWCNTs to remediate related pollutants in aqueous media via adsorption.

In the present study, the impacts of nonionic surfactant types and concentrations on PAH adsorption onto MWCNTs through adsorption kinetics and isotherms were assessed; furthermore, the adsorption mechanisms were elucidated through the changes in MWCNTs’ element compositions and structures before and after adding nonionic surfactants using TEM. This helps to increase the knowledge of MWCNTs used to adsorb toxic compounds, which is useful for the risk assessment of MWCNTs, and, at the same time, it can also help us to understand the role of MWCNTs in the migration and transportation of organic pollutants in the environment.

## 2. Materials and Methods

### 2.1. Materials

MWCNTs’ purity was higher than 95%; their outer diameter and length ranged from 20 to 30 nm and 10 to 30 μm, respectively. MWCNTs were purchased from XFNANO (Nanjing, China). MWCNTs were purified by HNO_3_ (65–68%) at 80 °C for 8 h. The mixtures were cooled naturally to around 25 °C and filtered using a suction filter device, and then washed with deionized water until the pH value of the filtrate was nearly 7.0. The dark precipitates were collected and warmed for 12 h at 80 °C in an oven (DGG-101-0, Tianjin, China). Finally, the dried MWCNTs were ground and kept in a desiccator for further use.

Phe was selected as a representative PAH and obtained from Acros Organics (Waltham, MA, USA) at 98% purity. A certain mass of Phe was dissolved in methanol to obtain 1 g/L of stock solution. Tween 80 (TW-80) and Triton X-100 (TX-100), purchased from Tianjin Jiangtian Chemical Technology Co., Ltd. (Tianjin, China), were selected as model nonionic surfactants.

### 2.2. Characterization of MWCNTs

Several techniques have been used for the characterization of MWCNTs. The C, H, and O content of MWCNTs were determined using a EA3000 Elemental Analyzer (LEEMAN, Genoa, Italy). Surface areas (SA) and pore volumes of MWCNTs were calculated from adsorption−desorption isotherms of N_2_ using a ASAP 2460 surface area analyzer (Micromeritics, Norcross, GA, USA). The structures of MWCNTs were characterized using an FEI Tecnai G2 F20 S-TWIN high-resolution transmission electron microscope (FEI, Hillsboro, OR, USA) with electron diffraction, operated at 300 kV. The morphology of MWCNTs was observed with a JSM-7800F (JEOL, Beijing, China) field-emission scanning electron microscope.

### 2.3. Adsorption Kinetics Experiments

#### 2.3.1. Adsorption Kinetics Experiment of Nonionic Surfactants and Phe by MWCNTs

Kinetic adsorption was carried out by batch experiments in 40 mL screw cap vial flasks at a given temperature (23 ± 1 °C). Adsorption experiments were conducted with 1 mg MWCNTs, 1 mg/L Phe, 0.01 mol/L CaCl_2_, and 200 mg/L NaN_3_ mixed with 40 mL solution containing different concentrations of TW-80 or TX-100, respectively. The vials were sealed with Teflon screw caps and shaken at 180 rpm at a given temperature (23 ± 1 °C). Then, the vials were centrifuged and the supernatant was taken out to measure the adsorption kinetics of the MWCNTs to Phe and the nonionic surfactant at given time intervals.

#### 2.3.2. Adsorption Kinetic Models

Lagergren pseudo-first-order model [31]:

(1) $$Q_{t}=Q_{e}\left(1-e^{-k_{1}t}\right)$$

Pseudo-second-order model [32]:

(2) $$\frac{t}{Q_{t}}=\frac{1}{k_{2}Q_{e}^{2}}+\frac{t}{Q_{e}}$$

Weber–Morris model [33]:

(3) $$Q_{t}=A+K_{a}t^{0.5}$$

where *t* (min) is the constant time; *k*_1_ (min^−1^) and *k*_2_ [g/(mg·min)] are the first-order and second-order equilibrium rate constants, respectively; *Qt* and *Qe* are the amounts of Phe or surfactants adsorbed to MWCNTs during adsorption time *t* and equilibrium (mg/g), respectively; the rate constant of *Ka* [mg/(g·h^−0.5^)] is from the Weber–Morris model; and *A* is a number related to the thickness of the interface.

### 2.4. Adsorption Isotherm Experiments

#### 2.4.1. Adsorption Isotherm Experiment of Phe by MWCNTs

For the Phe adsorption isotherm, 1 mg MWCNTs were mixed with 40 mL 0.1 to 1.2 mg/L of Phe solution, respectively. The pH value was adjusted to 7.0 using NaOH and HNO_3_ (1 mol/L) solutions, and then the mixtures were shaken at 160 rpm at 23 °C for 108 h. Then, the vials were kept still for 10 min and the supernatant was taken out to measure the residual Phe.

#### 2.4.2. Adsorption Isotherm Experiment of Phe by MWCNTs under the Influence of Nonionic Surfactants

For the adsorption isotherm of Phe by MWCNTs under the influence of nonionic surfactants, a 40 mL solution containing different concentrations of TW-80 or TX-100 was used, respectively. The following steps were the same as above.

#### 2.4.3. Adsorption Isotherm Models

Langmuir (Equation (4)) [34] and Frendlich (Equation (5)) [35] were employed to evaluate Phe adsorption by MWCNTs.

(4) $$\frac{C_{e}}{Q_{e}}=\frac{1}{K_{L}Q_{m}}+\frac{C_{e}}{Q_{m}}$$

(5) $$logQ_{e}=logK_{F}+\frac{1}{n}logC_{e}$$

where *C_e_* and *Q_m_* are the equilibrium Phe concentration in the aqueous phase and the maximum mass of Phe adsorption, respectively; *K_L_* and *K_F_* are the Langmuir and Freundlich isotherm parameters, respectively; and *n* is the heterogeneity parameter of the adsorbent MWCNT surface.

### 2.5. Phe and Nonionic Surfacant Analysis

The final concentrations of Phe in the supernatant were determined using HPLC (Agilent 1200, Frederick, CO, USA) and Venusil XBP C_18_ column (4.6 mm × 150 mm × 5 μm, 150 Å, Agela Technologies, Tianjin, China) analysis. During the measurement of Phe, the flow rate of the mobile phase at 90:10 (*v*/*v*) of methanol–water solution was 1.0 mL min^−1^.

The TW-80 and TX-100 were detected at 233 and 223 nm with an ultraviolet–visible (UV–visible) spectrophotometer (UV–Visible Spectrophotometer, UV-2450, SHIMADZU, Kyoto, Japan).

All experiments were performed in triplicate. The means and standard deviations were calculated by using SPSS 23.0.

## 3. Results and Discussion

### 3.1. Adsorption of Nonionic Surfactants

**Figure 1:**
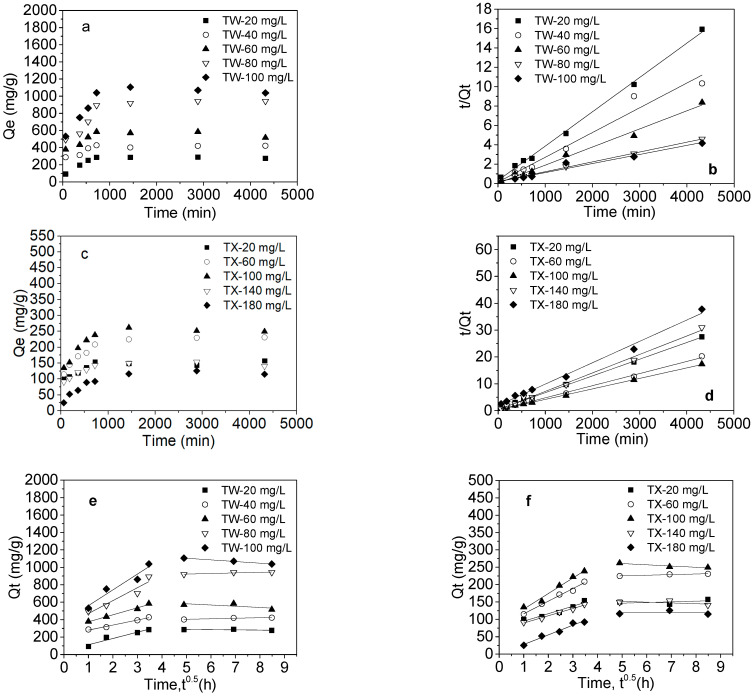
Adsorption kinetics of nonionic surfactants onto MWCNTs (**a**,**c**), pseudo-second-order model fitting (**b**,**d**), and Weber–Morris model fitting (**e**,**f**).

The adsorption kinetics of TW-80 and TX-100 onto MWCNTs are plotted in Figure 1. The adsorption of TW-80 and TX-100 onto MWCNTs was rapid in the first 12 h of contact time, and then achieved equilibrium at 72 h (Figure 1a,c). The initial steep adsorption curve suggested that the adsorption occurred rapidly on the surfaces of MWCNTs. Therefore, adsorption kinetic models of Lagergren pseudo-first-order kinetics and pseudo-second-order kinetics found in the literature are commonly used to describe the mechanisms of surfactant adsorption [36,37]. In this study, the adsorption kinetics of TW-80 and TX-100 onto MWCNTs were more suitable for the pseudo-second-order models, with higher *r*^2^ values ranging from 0.961 to 0.988 (Table 1). The theoretical values of *Q_Equation_* from the pseudo-second-order model were also closer to the experimental values of *Q_eq_*. The nonlinear adsorption kinetic equations indicated that the adsorption behaviors of TW-80 and TX-100 were related to the active adsorption sites on MWCNTs. TW-80 and TX-100 adsorption was better fitted with the pseudo-second-order kinetic model, which suggested that the adsorption mechanism of chemisorption between nonionic surfactants and the adsorbent occurred. Secondly, the main mechanism involved *π*–*π* interactions, hydrogen bonds, and hydrophobic bonds between the nonionic surfactants and MWCNTs for their adsorption [26,38,39]. Finally, the Weber–Morris model showed that *Q_t_* were linearly correlated with *t*^0.5^ (Table 1, Figure 1e,f), suggesting that the diffusion in pores is likely to not be a rate-controlling step for nonionic surfactants’ adsorption onto MWCNTs [40]. Therefore, a surface-diffusion mechanism possibly controlled the adsorption kinetics of nonionic surfactants. The diffusion rate of nonionic surfactants’ monomers from the boundary layer of liquid MWCNTs to the surfaces of MWCNTs determined their adsorption rate.

Table 1 shows that the second-order equilibrium rate constant (*k*_2_) first increased and then decreased with the increasing TW-80 concentration, and it obtained the maximum at the TW-80 concentration of 60 mg/L. The intercept values (*A*) from the Weber–Morris model at the TW-80 solution concentrations of 80 and 100 mg/L were 148.2 and 180.8, respectively, which were significantly higher than the intercept values (*A*) at low concentrations. This indicated that when the TW-80 concentration was higher than 60 mg/L, the great boundary layer effect reduced the adsorption rate of TW-80 from the solution to the MWCNTs’ surface. TW-80 adsorbed onto MWCNTs increased with the increasing concentration. Surfactant adsorption from the solution to the MWCNTs’ surface depended on their concentration [41]. The TW-80 mainly existed as monomers when its concentration was below 13–15 mg/L, whereas TW-80 formed micelles when its concentration was above 15 mg/L, because the added surfactant molecules were easily aggregated [42]. There was an equilibrium relationship between the micelles and monomers of the surfactant in the solutions. When the surfactant was adsorbed from the solution to the surface of the MWCNTs and formed self-associated hemi-micelle aggregates on the solid surface, the equilibrium between the micelles and the monomer was disturbed, resulting in the dissociation of the micelles [43].

For TX-100, the adsorption rate constant (*k*_2_) was the maximum at a TX-100 concentration of 140 mg/L and then decreased rapidly (Table 1). Hence, the adsorption rate will decrease at high concentrations. Similarly, the adsorption amount of TX-100 onto MWCNTs also increased first and then decreased when the concentration of TX-100 increased. This may be because, when the TX-100 concentrations were 140 and 180 mg/L, which were also close to or higher than CMC 150 mg/L [44], the surfactant spontaneously formed self-associated micelles. However, MWCNT bundles that are close to each other cannot match well between the two. This caused MWCNTs to form aggregates due to osmotic pressure; the inner surface of the MWCNTs’ tube bundle will not adsorb the surfactant, resulting in a decrease in the amount of TX-100 adsorption. Our study proved that the adsorption of TX-100 onto MWCNTs had obvious differences from that of TW-80.

### 3.2. Adsorption Kinetics of Phe on MWCNTs as Affected by Nonionic Surfactants

**Figure 2:**
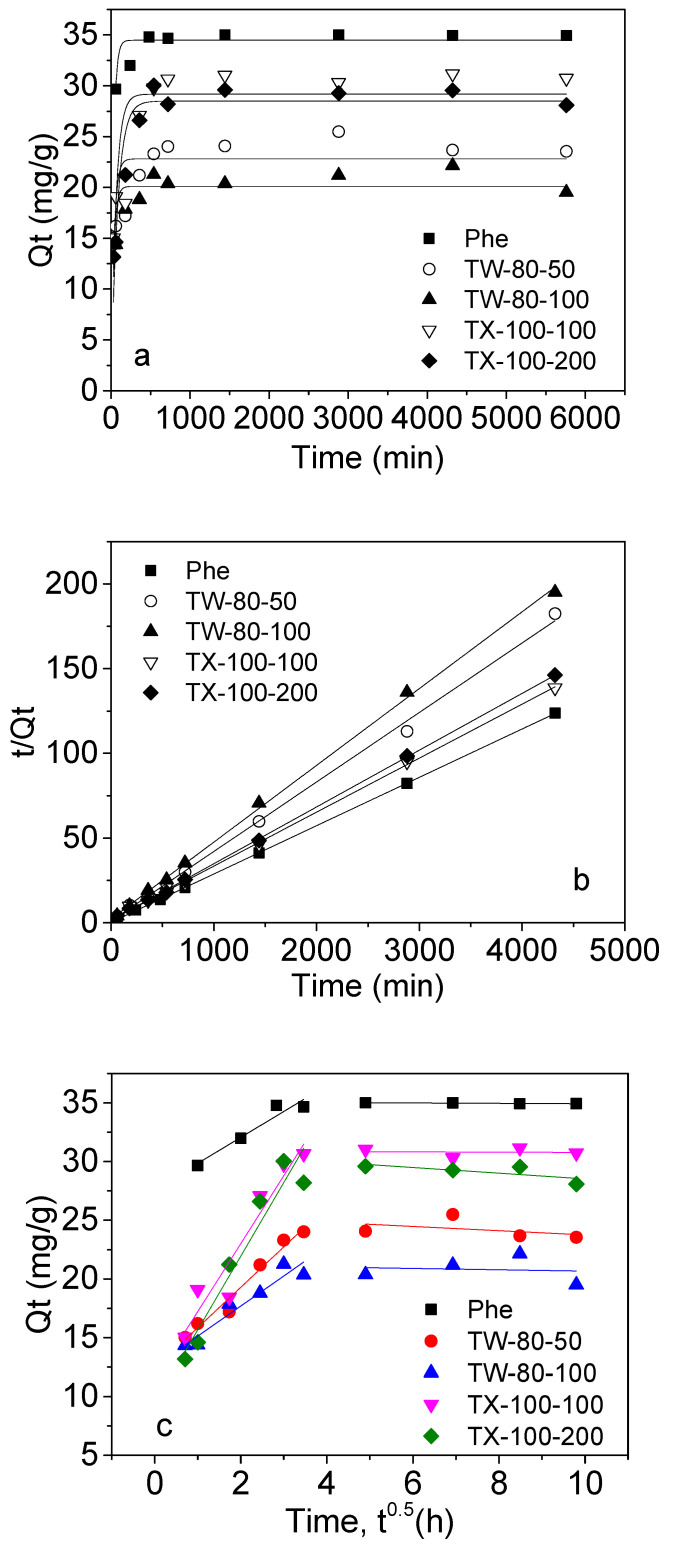
Non-linear fitting of pseudo-first-order model (**a**) and pseudo-second-order model (**b**), and plots of Weber–Morris diffusion model (**c**) for the adsorption of phenanthrene onto MWCNTs.

The effects of surfactants on the adsorption kinetics of Phe onto MWCNTs are presented Figure 2. The adsorption rate of Phe increased rapidly at the initial stage; thereafter, it increased slowly and obtained equilibrium at 24 h. Moreover, 87.3% of Phe was adsorbed on the MWCNTs when the adsorption time was 96 h. However, the surfactants’ addition decreased the adsorption rate of Phe onto MWCNTs. For example, the adsorption rate declined to 58.9% and 54.8% after adding 50 and 100 mg/L of TW-80, respectively, while the rate decreased to 76.8% and 73.3% when the TX-100 concentrations were 100 and 200 mg/L.

In general, the removal mass for Phe using MWCNTs as affected by surfactants was in the order MWCNTs > TX-100-100 + MWCNTs > TX-100-200 + MWCNTs > TW-80-50 + MWCNTs > TW-80-100 + MWCNTs. Therefore, the TW-80 more seriously prevented the Phe from being adsorbed onto MWCNTs than TX-100.

The adsorption mechanisms and the potential rate-controlling step were evaluated using the kinetic models. The theoretical *Q_Equation_* values for Phe that were calculated from the pseudo-second-order rate model were very close to the experimental *Q_Equation_* values (Table 2). Additionally, it was concluded from the *R*^2^ values shown in Table 2 that the experimental data better fitted to the pseudo-second-order kinetic model than the pseudo-first-order kinetic model. Thus, these results suggested that the pseudo-second-order adsorption played the main role in the Phe adsorption mechanism.

The kinetic adsorption parameters showed that the adsorption rate constant (*k*_2_) of Phe as affected by surfactants was in the order MWCNTs > TW-80-50 + MWCNTs > TX-100-200 + MWCNTs > TX-100-100 + MWCNTs > TW-80-100 + MWCNTs (Table 2). We concluded from the *k*_2_ parameter that TW-80-50 had lower hindrance than that of other surfactant conditions when approaching and further interacting with MWCNTs. In fact, the order of the removal capacity for Phe by MWCNTs as affected by surfactants was not consistent with the order of *k*_2_. Therefore, it was still uncertain whether the diffusion process of Phe molecules from the boundary of liquid MWCNTs to MWCNTs’ surfaces or in diffusion into the particles controlled their adsorption rate to MWCNTs as affected by surfactants.

The higher *R*^2^ values in the Weber–Morris model further indicated that the adsorption data of Phe by MWCNTs were better matched with the external diffusion of the first stage (Table 2). As reported, when *Q*_t_ against *t*^0.5^ was plotted through the origin using a single linear regression line, intraparticle diffusion determined the rate-controlling step [45,46]. Figure 2c shows the multi-linearity plots, indicating that the *Q*_t_ of Phe adsorption onto MWCNTs had two linear regressions, suggesting that intraparticle diffusion may not be a rate-controlling step for Phe adsorbed to MWCNTs. The first stage is the instantaneous stage with the initial sharp adsorption, and the diffusion rate of Phe from the boundary of liquid MWCNTs to the surfaces of MWCNTs determined their adsorption rate. This may be due to the rapid movement of Phe molecules in the aqueous phase under shaking, which greatly promoted their approach to the boundary of liquid MWCNTs; then, the Phe molecules moved to the external surfaces of MWCNTs. Additionally, the surface diffusion mechanism depended on the surfactant types and concentrations when comparing the adsorption rate constant (*K*_a_) (Table 2), because TW-80 and TX-100 had different molecular weights. The second adsorption stage occurred on the interior surfaces of MWCNTs; the equilibrium stage was controlled by intraparticle diffusion, and the Phe molecules slowly diffused into the micropores [46]. Additionally, the external diffusion model fitted the experimental data well, which indicated that the adsorption mechanism of Phe as affected by TW-80 and TX-100 was not complex, and the Phe adsorbed onto MWCNTs was controlled by external and intraparticle diffusion.

### 3.3. Equilibrium Adsorption of Phe on MWCNTs as Affected by Nonionic Surfactants

**Figure 3:**
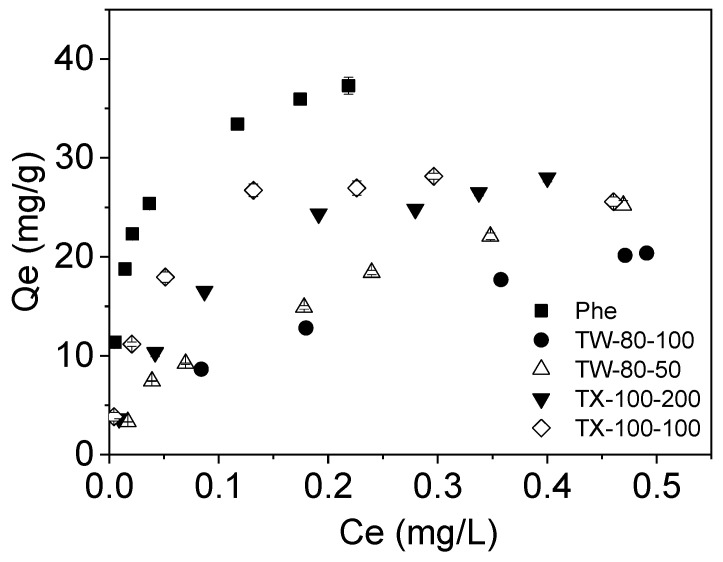
Adsorption isotherms of phenanthrene onto MWCNTs affected by nonionic surfactants.

To better elucidate the effect of surfactants on the Phe adsorption onto MWCNTs, the adsorption Freundlich and Langmuir isotherms were further studied, as shown in Figure 3 and Table 3. As shown in Table 3, the correlation coefficients (*R^2^*) ranged from 0.897 to 0.999 and from 0.987 to 0.998 as derived from the linear regression of the Freundlich and the Langmuir models, respectively. All isotherms of Phe were nonlinear and fitted well by the Freundlich and Langmuir models. Overall, the Langmuir model was more suitable than the Freundlich model in most cases according to their corresponding *R^2^* values. Therefore, it can be judged that the adsorption of Phe on MWCNTs is mainly based on monolayer adsorption, but it is not completely due to monolayer adsorption. Yang et al.’s (2006) research also showed that PAHs adsorbed onto the carbon nanomaterials neither formed a monolayer on a homogeneous surface nor formed a simple multilayer, which is consistent with our speculations. However, Gotovac et al. [47] indicated that Phe formed a multilayer physical adsorption film on the surface of single-wall carbon nanotubes (SCWNTs), which is inconsistent with our results. It may be that the SWCNTs are single-layer graphite layers, and it is easy to form a plurality of layers on the surface of the Phe. MWCNTs are composed of multiple layers of graphite with a small interlayer distance, which is not conducive to organic molecules’ entry, and it is also difficult to form multiple layers on the outer surface. The saturated adsorption capacity in the Langmuir model is lower than the actual adsorption capacity. The data in Table 4 also show that the MWCNTs were mainly composed of macropores and mesopores, and contained a small amount of micropores. The Phe molecule is small enough to occupy the surface sites in micropores. Combined with the adsorption kinetics results, the adsorption of Phe on MWCNTs was mainly surface adsorption, and a small amount of Phe entered the pores of MWCNTs.

**Figure 4:**
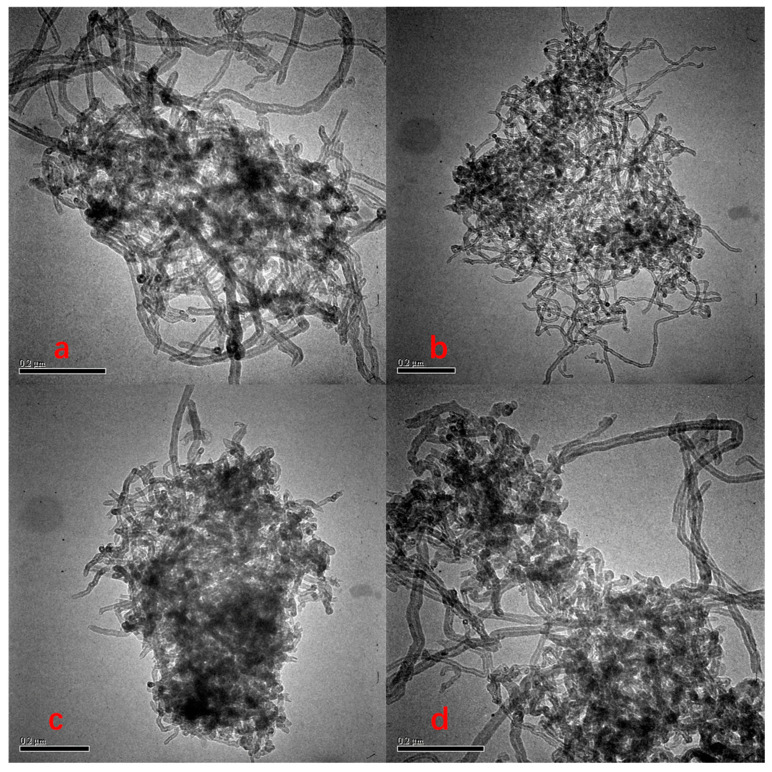

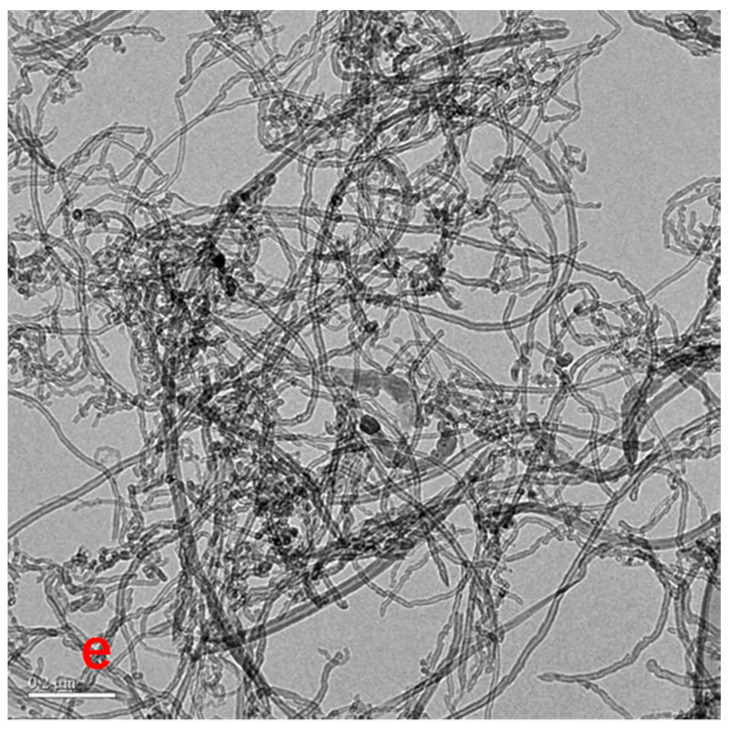
TEM of MWCNTs on TW-80 interacting with MWCNTs (**a**,**b**), TX-100 interacting with MWCNTs (**c**,**d**), and MWCNTs (**e**).

The adsorption nonlinearity of Phe on MWCNTs was weakened in the presence of nonionic surfactants, and both nonionic surfactants inhibited the adsorption of Phe on MWCNTs. When TW-80 and TX-100 were present in the aqueous phase, the saturated adsorption capacity of Phe decreased from 35.97 mg/g to 27.10 and 29.79 mg/g, respectively. The reasons might be threefold. Firstly, though surfactants could diffuse carbon nanotubes [48], surfactants adsorbed onto MWCNTs (Figure 1) reduced the specific surface area of MWCNTs (Table 4). The order of the specific surface area of MWCNTs after the adsorption of nonionic surfactants was MWCNTs > TW-80-50 + MWCNTs > TW-80-100 + MWCNTs > TX-100-100 + MWCNTs > TX-100-200 + MWCNTs. TEM images of MWCNTs (Figure 4) showed that the accessible surface area and porosity for the adsorption of MWCNTs were reduced by the strong aggregation of MWCNTs, which further reduced the Phe adsorption. At the same time, studies have shown that the interaction of MWCNTs with Phe is mainly hydrophobic, with hydrogen bonding and π−π interaction [49]. Table 4’s data prove that when surfactants were adsorbed onto the surfaces of MWCNTs, the C element of MWCNTs decreased, the O element and H element increased, and the polarity increased, which led to the weakening of the hydrophobic interactions between MWCNTs and Phe.

Second, our results showed that TW-80 and TX-100 could be adsorbed onto the MWCNTs, which indicated that surfactants occupied the sites adsorbing Phe. Therefore, surfactants produce competitive adsorption sites on carbon nanomaterials [50]. Yang et al. (2006) indicated that the adsorption nonlinearity of the main solute is weakened by a coexisting substance in the same adsorption system. Our result is consistent with those of the adsorption of Phe on multilayer graphene as affected by the surfactant and exfoliation [51].

Third, the Phe molecule in the MWCNT–water system may be present in the micelle or aqueous solution, directly adsorbed to the carbon nanotube, or adsorbed to the adsorbed surfactant on the MWCNTs. The surfactant can desorb a large amount of the adsorbed Phe on the surface of the MWCNTs. This can explain why the specific surface area of MWCNTs after the adsorption of nonionic surfactants was inconsistent with the ability to adsorb Phe. The Phe adsorption capacities were decreased more when surfactants were present in higher concentrations (Figure 3). When the surfactant concentrations were higher, more surfactant micelles would remain in the aqueous state, enhancing the solubility or desorption of more PAHs and furthermore decreasing PAH adsorption [41]. The saturated adsorption capacity of Phe on MWCNTs affected by surfactants was MWCNTs > TX-100-100 + MWCNTs > TX-100-200 + MWCNTs > TW-80-50 + MWCNTs > TW-80-100 + MWCNTs (Table 2). The higher the TW-80 concentration, the more micelles were formed; moreover, the Phe molecules were wrapped in the hydrophobic center of the micelles, and the hydrophilic surface of the micelles was evenly distributed throughout the aqueous phase, resulting in the high apparent solubility of the Phe. Therefore, the TW-80 inhibition effect was stronger, and the high-concentration inhibition was higher than that at the low concentration. The high concentration of TX-100 inhibition was higher than at the low concentration. The CMC of TX-100 is 150 mg/L, and the value for TX-100-100 was lower than the CMC, so it could not form micelles. There may still be adsorption sites on the surfaces of MWCNTs. While TX-100-200 was higher than the CMC, the surfaces of carbon nanotubes adsorbed more TX-100 molecules, and Phe was solubilized in the micelle shells.

When the concentration of surfactant is higher than its critical micelle concentration, the surfactant exists in the MWCNT–water system as monomers and micelles, and is adsorbed on the MWCNTs in a monomolecular state and hemi-micelle state [52]. The sizes of nonionic surfactant micelles and Phe molecules in water were in the order of TX-100 (20 nm) > TW-80 (10 nm) > Phe (0.6 nm) [26,53,54], respectively. Surfactant molecules/micelles of different sizes were adsorbed on the surfaces of MWCNTs, reducing the surface area of MWCNTs and blocking the micropores of MWCNTs [41,55]. The SA and pore volume of MWCNTs after adsorbing large-sized TX-100 molecules/micelles were significantly smaller than those after adsorbing small-sized TW-80 molecules/micelles (Table 4). The increase in average pore diameter and the disappearance of micropores of MWCNTs indicated fewer adsorption sites available for Phe adsorption (Table 4), and the presence of nonionic surfactants significantly inhibited the adsorption of Phe onto MWCNTs (Table 2 and Table 3). Moreover, TX-100 with a larger micelle size not only had a lower adsorption capacity on MWCNTs than TW-80 at the same concentration, but also introduced more steric effects and effectively dispersed MWCNTs (Table 1) (Bai 2010). Therefore, TX-100 had a weaker inhibitory effect on MWCNTs adsorbing Phe than TW-80 (Table 3). The experimental results showed that the adsorption amount of polyoxyethylene nonionic surfactant on the sediment was positively correlated with the length of the polyoxyethylene chain in its molecular structure [49], which was one of the reasons for the weaker inhibition of TX-100.

**Figure 5:**
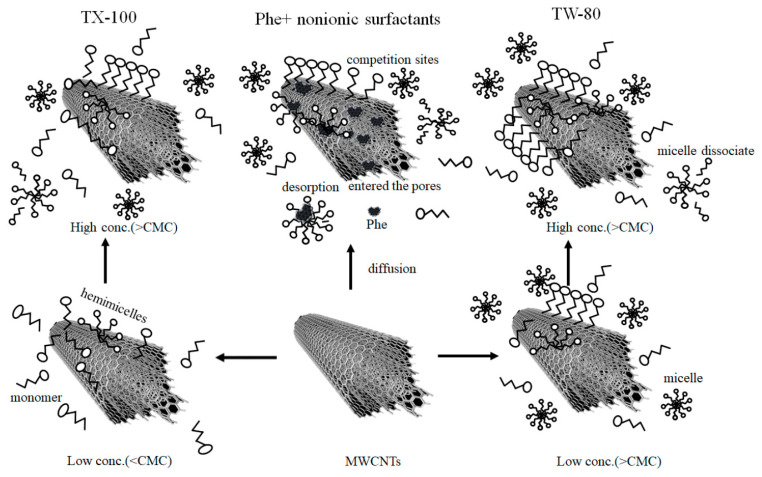
Scheme of adsorption mechanisms of phenanthrene adsorbed onto MWCNTs as affected by nonionic surfactants.

When Phe and nonionic surfactant solutions are mixed with MWCNTs, the proposed adsorption scheme is as shown in Figure 5. In the first step, the Phe and nonionic surfactant monomers diffused and went through the liquid film to the external surface of the MWCNTs. When micelles were present in the solution, Phe was dissolved in the micelles’ core and shells. In the second step, the Phe and nonionic surfactant monomers were absorbed on the MWCNT interface. The different concentrations of nonionic surfactants are adsorbed on the surfaces of MWCNTs in different forms of hemi-micelles or multilayer [41], and compete with Phe for adsorption sites on the surfaces of MWCNTs, preventing Phe from entering micropores. In the third step, at concentrations greater than the CMC, micelles dissociate due to adsorption to restore equilibrium, and Phe is released from micelles and enters the adsorbed surfactant micelles. At the same time, newly formed micelles may also desorb Phe from the surfaces of MWCNTs. In the fourth step, the Phe adsorbed on the surfaces of the MWCNTs slowly moves into the micropores.

## 4. Conclusions

The results on the effect of TW-80 and TX-100 on the adsorption of Phe onto MWCNTs showed that TW-80 and TX-100 can be well adsorbed by MWCNTs, and their adsorption kinetics were fitted with the pseudo-second-order model and Weber–Morris model. When the concentration of TW-80 is greater than the CMC, TW-80 adsorption onto MWCNTs increases with the increasing concentration. This is because the TW-80 micelles dissociate to form more monomers. When TX-100 is at a low concentration, TW-80 adsorption onto MWCNTs also increases with the increasing concentration. However, when the concentration of TX-100 is close to or higher than the CMC, the adsorption of TX-100 decreases due to the enhanced hydrophilic interaction and the aggregation of carbon nanotubes. The experimental kinetic data for Phe adsorption by MWCNTs all worked well with the pseudo-second-order kinetics before and after TW-80 and TX-100. Phe adsorbed onto MWCNTs was controlled by external and intraparticle diffusion, which was confirmed by the Weber–Morris model. The adsorption capacities of Phe onto MWCNTs were inhibited by TW-80 and TX-100, which were the possible reasons that, after MWCNTs adsorbed the surfactants, the specific surface area decreased, the micropores disappeared, the polarity increased, and the nonionic surfactants competed with Phe for adsorption sites and could promote the desorption of Phe. The study confirms the adsorption mechanism by which nonionic surfactants affect organic pollutants adsorbed onto MWCNTs.

## Figures and Tables

**Table 1:** Parameters of pseudo-first-order and pseudo-second-order model and Weber–Morris model for adsorption kinetics of nonionic surfactants onto MWCNTs.

Surfactant	Pseudo-First-Order Model			Pseudo-Second-Order Model			Weber–Morris Model		
	*Q_e_* (mg·g^−1^)	*k*_1_ (min^−1^ × 10^−3^)	*R* ^2^	*Q_e_* (mg·g^−1^)	*k*_2_ (g·mg^−1^·min^−1^ × 10^−5^)	*R* ^2^	*A*	*Ka* (mg·g^−1^·h^0.5^)	*R^2^*
TW-80-20	284.568	4.060	0.932	281.690	3.985	0.997	72.152	40.032	0.902
TW-80-40	396.186	21.37	0.433	390.625	4.288	0.962	57.658	222.344	0.980
TW-80-60	535.288	20.290	0.465	534.760	12.075	0.988	80.383	294.680	0.982
TW-80-80	916.562	3.680	0.344	980.392	0.513	0.993	148.240	321.840	0.867
TW-80-100	985.475	11.200	0.588	1067.027	0.455	0.961	180.776	380.236	0.900
TX-100-20	140.600	16.390	0.385	160.771	7.620	0.999	73.067	21.634	0.922
TX-100-60	212.530	7.380	0.656	221.730	6.953	0.997	79.726	36.324	0.984
TX-100-100	241.890	7.080	0.667	255.755	5.006	0.998	84.753	44.830	0.973
TX-100-140	136.409	14.24	0.382	145.560	14.656	0.993	68.368	20.977	0.985
TX-100-180	118.959	1.590	0.960	125.786	3.225	0.991	0.243	27.833	0.964

**Table 2:** Parameters of pseudo-first-order and pseudo-second-order model and Weber–Morris model for adsorption kinetics of Phe onto MWCNTs as affected by surfactants.

Compound			Pseudo-First-Order Model			Pseudo-Second-Order Model			Weber–Morris Model		
		*Q_e_*, exp (mg·g^−1^)	*Q_e_* (mg·g^−1^)	*k*_1_ (min^−1^ × 10^−2^)	*R* ^2^	*Q_e_* (mg·g^−1^)	*k*_2_ (g·mg^−1^·min^−1^ × 10^−4^)	*R* ^2^	*A*	*K_a_* (mg·g^−1^·h^0.5^)	*R* ^2^
Phe	MWCNTs	34.930	34.400	3.296	0.680	35.051	29.095	0.999	27.640	2.210	0.896
	TW-80-50	21.890	22.804	2.683	0.533	24.378	15.735	0.997	12.400	3.440	0.965
	TW-80-100	23.560	20.093	3.107	0.587	22.065	8.945	0.999	12.570	2.560	0.899
	TX-100-100	29.320	29.172	1.623	0.629	31.387	6.359	0.998	11.420	5.790	0.905
	TX-100-200	30.720	28.483	1.219	0.865	29.842	8.168	0.999	9.380	6.270	0.913

**Table 3:** Langmuir and Freundlich and dual model parameters for the adsorption of surfactants and Phe onto MWCNTs under TW-80 and TX-100 conditions.

Compound		Freundlich				Langmuir	
		*k*	1/*n*	*R* ^2^	*Q_m_* (mg·g^−1^)	*A*	*R* ^2^
Phe	MWCNT	59.501	0.268	0.960	35.793	0.012	0.987
	TW-80-100	29.996	0.503	0.250	27.102	0.182	0.994
	TW-80-50	48.911	0.609	0.671	28.490	0.125	0.992
	TX-100-200	62.359	0.597	0.962	29.790	0.065	0.996
	TX-100-100	84.092	0.563	0.912	30.990	0.029	0.998

**Table 4:** Bulk elemental composition and pore volume of MWCNTs.

	Bulk Elemental Composition (%)							Pore Volume (cm^3^/g)	
	C	H	O	N	ash	SA (m^2^/g)	Pore size (nm)	Vmic	Vmes + mac
MWCNTs	95.975	0.100	1.809	0.984	1.132	142.048	22.764	0.012	0.796
TW-80-60	94.598	0.669	2.320	0.906	1.507	116.673	31.013	0	0.905
TW-80-100	93.271	0.783	2.800	0.100	3.046	109.420	31.070	0	0.850
TX-100-100	93.342	0.883	3.556	0.889	1.330	89.537	34.682	0	0.705
TX-100-180	89.901	1.101	4.111	0.779	4.108	76.719	31.484	0	0.665

## Data Availability

All the research data have been included in the manuscript, others if any, can be available from the corresponding author upon reasonable request.
